# Room temperature energy-efficient spin-orbit torque switching in two-dimensional van der Waals Fe_3_GeTe_2_ induced by topological insulators

**DOI:** 10.1038/s41467-023-40714-y

**Published:** 2023-08-24

**Authors:** Haiyu Wang, Hao Wu, Jie Zhang, Yingjie Liu, Dongdong Chen, Chandan Pandey, Jialiang Yin, Dahai Wei, Na Lei, Shuyuan Shi, Haichang Lu, Peng Li, Albert Fert, Kang L. Wang, Tianxiao Nie, Weisheng Zhao

**Affiliations:** 1 Fert Beijing Institute, MIIT Key Laboratory of Spintronics, School of Integrated Circuit Science and Engineering, Beihang University, Beijing, China; 2 Shenyuan Honors College, Beihang University, Beijing, China; 3 Department of Electrical and Computer Engineering, and Department of Physics and Astronomy, University of California, Los Angeles, CA USA; 4 State Key Laboratory of Superlattices and Microstructures, Institute of Semiconductors, Chinese Academy of Sciences, P.O. Box 912 Beijing, China; 5 Department of Electrical and Computer Engineering, Auburn University, Auburn, Alabama USA; 6 Unité Mixte de Physique, CNRS, Thales, Univ. Paris-Sud, University of Paris-Saclay, Palaiseau, France

**Keywords:** Topological insulators, Magnetic properties and materials, Two-dimensional materials, Spintronics

## Abstract

Two-dimensional (2D) ferromagnetic materials with unique magnetic properties have great potential for next-generation spintronic devices with high flexibility, easy controllability, and high heretointegrability. However, realizing magnetic switching with low power consumption at room temperature is challenging. Here, we demonstrate the room-temperature spin-orbit torque (SOT) driven magnetization switching in an all-van der Waals (vdW) heterostructure using an optimized epitaxial growth approach. The topological insulator Bi_2_Te_3_ not only raises the Curie temperature of Fe_3_GeTe_2_ (FGT) through interfacial exchange coupling but also works as a spin current source allowing the FGT to switch at a low current density of ~2.2×10^6^ A/cm^2^. The SOT efficiency is ~2.69, measured at room temperature. The temperature and thickness-dependent SOT efficiency prove that the larger SOT in our system mainly originates from the nontrivial topological origin of the heterostructure. Our experiments enable an all-vdW SOT structure and provides a solid foundation for the implementation of room-temperature all-vdW spintronic devices in the future.

## Abstract (web summary)

Magnetic random access memory (MRAM) exhibits remarkable device endurance, while also offering potential operation speed and energy efficiency improvements compared to conventional random access memory. However, challenges remain, both in terms of efficiency, and miniaturization. Here, Wang et al demonstrate a van der Waals (vdW) based spin-orbit torque switching, in a Fe_3_GeTe_2_/Bi2Te_3_ heterostructure, paving the way for thinner and higher efficiency spin-orbit torque based vdW MRAM.

## Introduction

Spin-transfer torque^1–5^ magnetic random access memory (STT-MRAM) is an appealing alternative to overcome the performance bottleneck encountered in traditional semiconductor-based memory, offering superior performance in terms of nonvolatility, high density, and low-power dissipation. However, the initiative parallel or antiparallel collinear magnetic configuration would lead to an incubation delay when using STT for magnetic switching, and the large writing current could break down the tunneling barrier. In comparison, spin–orbit torque (SOT)^6–10^ could eliminate such performance drawbacks and allow for faster operation, better endurance, and higher energy efficiency. Therefore, it is of fundamental and technical importance to use SOT for switching the magnetization, which is expected to become the major competitor for next-generation memories^11–15^.

To date, great efforts have been devoted to exploring new principles and materials for realizing high-performance SOT devices^16–20^. Usually, heavy metals that include W, Ta, Pt, etc., were employed as the spin current sources through charge-spin conversion, which could exert a torque on the adjacent ferromagnetic layer for magnetization switching^21–24^. For higher SOT efficiency, van der Waal (vdW) topological insulators (TIs) were recently suggested as a replacement for heavy metals due to their unique feature of spin-momentum locking in the non-trivial topological surface state (TSS). This has been demonstrated to allow for high-efficiency SOT-driven magnetic switching in three-dimensional (3D) ferromagnet at room temperature with low critical switching current^17,25–28^. However, 3D ferromagnets would limit the size scaling and lower the spin transparency due to the dangling bonding interface^29^. Therefore, there is a need to develop new material systems with lower dimensions and superior interfaces for higher SOT efficiency^30–32^, which may bring new opportunities to break the power consumption bottleneck of integrated circuits^33^.

The recently-discovered vdW 2D ferromagnetic materials offer an atomic flat surface and can maintain their magnetic ordering down to the 2D limit, which would satisfy such demand. The Mermin–Wagner–Hohenberg (MWH) theorem predicted that thermal fluctuations in a 2D magnetic system^34,35^ forbade the long-range magnetic order at finite temperature because the continuous symmetry could not be spontaneously broken in a 2D system. However, recently it has been discovered that 2D intrinsic ferromagnetic materials could exist through breaking the MWH theorem by magnetic anisotropy such as Fe_3_GeTe_2_ (FGT)^36^, CrI_3_^37^, and Cr_2_Ge_2_Te_6_^38^, among others. FGT has received extensive attention by virtue of its hard magnetic properties, Kondo lattice behavior, itinerant ferromagnetism, and other fascinating characteristics^39–43^. Remarkably, intercalating lithium ions into the interlayer gap of FGT can change the density of states on the Fermi surface and successfully raise the *T*_*c*_ to room temperature^44^. These inspiring results suggest FGT is an ideal 2D candidate for exploring SOT-driven-magnetic switching. Recently, the SOT-driven magnetization switching of FGT has been demonstrated using Pt as a spin current source through the spin Hall effect or interfacial Rashba–Edelstein effect^45,46^. However, these devices only work at low temperatures (<200 K). Furthermore, much higher SOT efficiency could be envisaged through constructing the all-vdW heterostructure, which can provide a clean interface and thus support high interfacial spin transparency. Therefore, there is an urgent need to design all-vdW heterostructures to achieve energy-efficient SOT switching that can operate at room temperature for future 2D spintronic applications.

Here, we realize SOT-driven magnetic switching in an MBE-grown all-vdW Bi_2_Te_3_/FGT heterostructure at room temperature. The SOT-induced magnetization switching is achieved with a critical switching current density of ~2.2 × 10^6^ A/cm^2^. The damping-like SOT efficiency was calculated to be about ~2.69 at room temperature. The high efficiency proves the superior characteristics of all-vdW heterostructures constructed from 2D ferromagnetic materials. We analyze the difference between the large-field power-law fitting and the small-field derivation fitting from the harmonic measurements. In particular, the weak vdW interactions between adjacent layers make it possible to combine atomic layers with different matching degrees, thereby getting rid of lattice matching and compatibility restrictions. The high-quality heterostructure interface is one of the most important factors for achieving high spin transmissivity. Our results provide a paradigm for the construction of all-vdW SOT devices at room temperature and promote the development of 2D ferromagnets for practical applications.

## Results

### Magneto-transport measurements in Bi_2_Te_3_, FGT, and Bi_2_Te_3_/FGT heterostructures

**Fig. 1:**
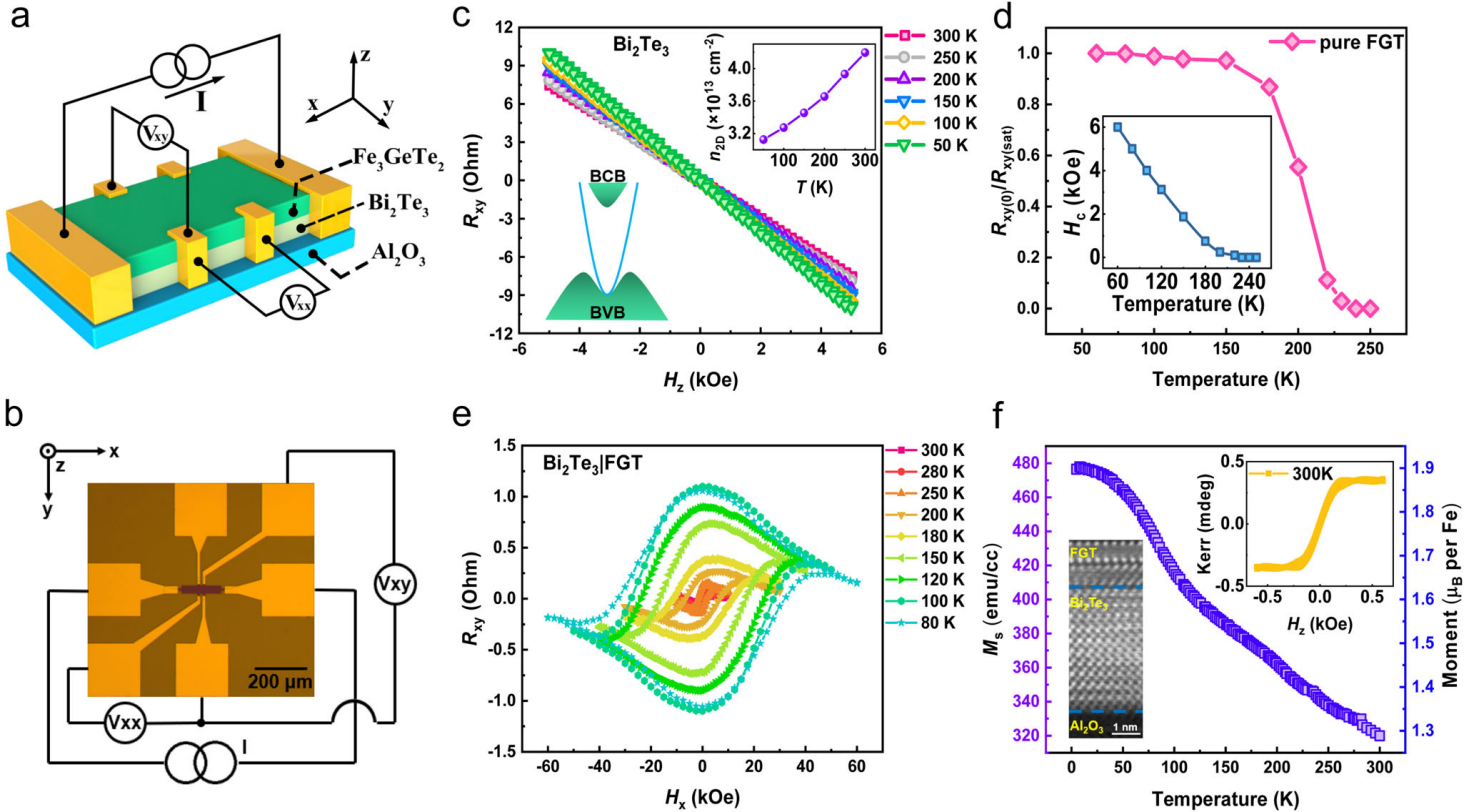
Electrical and magnetic measurements in Bi_2_Te_3_, Fe_3_GeTe_2_, and Bi_2_Te_3_/Fe_3_GeTe_2_. **a** Schematic diagram of the device measurement setup. **b** Optical micrograph of a Hall bar device for electric measurement. **c** *R*_*xy*_–*H*_*z*_ curves at different temperatures in 8 nm Bi_2_Te_3_. The left inset shows the schematic of the band structure, and the right inset shows the temperature-dependent carrier density. **d** Normalized remnant anomalous Hall resistance and coercivity as a function of temperature in pure FGT, which displays the Curie temperature is ~220 K. **e** *R*_*xy*_ as a function of in-plane field *H*_*x*_ at different temperatures in Bi_2_Te_3_(8)/FGT(3) heterostructure, which displays the perpendicular magnetic anisotropy. **f** Curves of saturation magnetization *M*_s_ at different temperatures in Bi_2_Te_3_/FGT heterostructure. The right inset displays the Kerr signal of the heterostructure at 300 K, and the left inset displays the crystalline quality by HAADF-STEM image.

In this work, we deposited thin films on a (0001) sapphire substrate by MBE, combined with the reflection high-energy electron diffraction (RHEED) to in situ monitor the surface structure of the film during the preparation, and analyzed the surface morphology by atomic force microscopy (AFM). When preparing the wafer-scale all-vdW heterostructure, it is very critical to maintain the surface flatness of the bottom layer to ensure the optimal lattice matching and compatibility of the two layers. Therefore, after growing topological insulators on (0001) sapphire substrates, the growth temperature needs to be slowly increased in the growth chamber to maintain a Te-rich environment, which will ensure an excellent single crystallinity of the heterostructure. To better understand the sample quality, RHEED was in situ rotated during the growth process to check the in-plane crystallinity. During the rotation, RHEED stripes changed regularly and coherently, which could exclude the presence of multidomain. To prevent its degradation, we covered the top surface of FGT with a protective layer. Micrometer-sized Hall-bar devices were fabricated by the standard photolithography combined with ion beam etching. The schematic of the device and the measurement setup are shown in Fig. 1a. The Hall-bar structure was patterned with the dimensions of 100 μm (length) × 30 μm (width) for electrical transport measurements, as shown in Fig. 1b, where *V*_*xy*_ and *V*_*xx*_ represent the Hall and longitudinal voltage, respectively. As an emergent quantum matter, TIs attract a lot of interest due to the bulk gap and the spin-momentum-locked Dirac fermions on the surface. Hence, for these types of materials, such as Bi_2_Te_3_ and Bi_2_Se_3_, it has been proved by both the theory and experiment that surface states consist of a single Dirac cone at the Γ point, and its simplicity has become an ideal object for studying the spintronics and electronics simultaneously. In the following, we grew 8 nm Bi_2_Te_3_ on the sapphire substrate and performed the magneto-transport measurement. By applying an out-of-plane magnetic field, the Hall resistance shows a negative slope, as shown in Fig. 1c, which features the n-type Bi_2_Te_3_. The right inset in Fig. 1c presents the temperature-dependent 2D carrier density (*n*_2D_)^47^ obtained from the Hall data, which reflects the conduction dominantly from the bulk state. The left inset of Fig. 1c elucidates the band structure of Bi_2_Te_3_, and the position of the Fermi level (*E*_F_) determines the spin-momentum locking properties. As a layered vdW crystal, FGT has metallic ferromagnetism^36,41^. Each vdW layer is composed of five atomic sublayers with the lattice constants of *a* = *b* = 3.9536 (7) Å and *c* = 16.396 (2) Å. During the growth of FGT in the MBE chamber, the stripe-like RHEED pattern was captured, reflecting an atomic smooth interface, and its crystal structure was further characterized by X-ray diffraction (XRD) (Supplementary Fig. S1). To clarify the magnetic behavior of FGT, we conducted magneto-transport measurements by a physical property measurement system (PPMS) on a 30 nm FGT thin film. Figure 1d clearly shows the temperature-driven transition from a ferromagnetic to a paramagnetic state with the *T*_*c*_ around 220 K, which is the same as that in the previous report^44^. Figure 1e shows the hysteresis loops between 80 K and 300 K in Bi_2_Te_3_(8)/FGT(3) heterostructure with an in-plane magnetic field, which verified its perpendicular magnetic anisotropy (PMA) feature. The number enclosed in brackets denotes the thickness of the individual layer in nanometers. Furthermore, the saturation magnetization (*M*_*s*_) and the magnetic properties in Bi_2_Te_3_(8)/FGT(3) were characterized by a superconducting quantum interference device (SQUID) in Fig. 1f, clearly showing the room temperature ferromagnetism. The fascinating phenomenon of the combination of topological insulators and magnetic 2D materials lays the foundation of our current research. The high-angle annular dark-field scanning transmission electron microscopy (HAADF-STEM) in the inset further confirms the atomic structure of Bi_2_Te_3_ and FGT.

### Current-induced SOT switching in Bi_2_Te_3_/FGT heterostructure

**Fig. 2:**
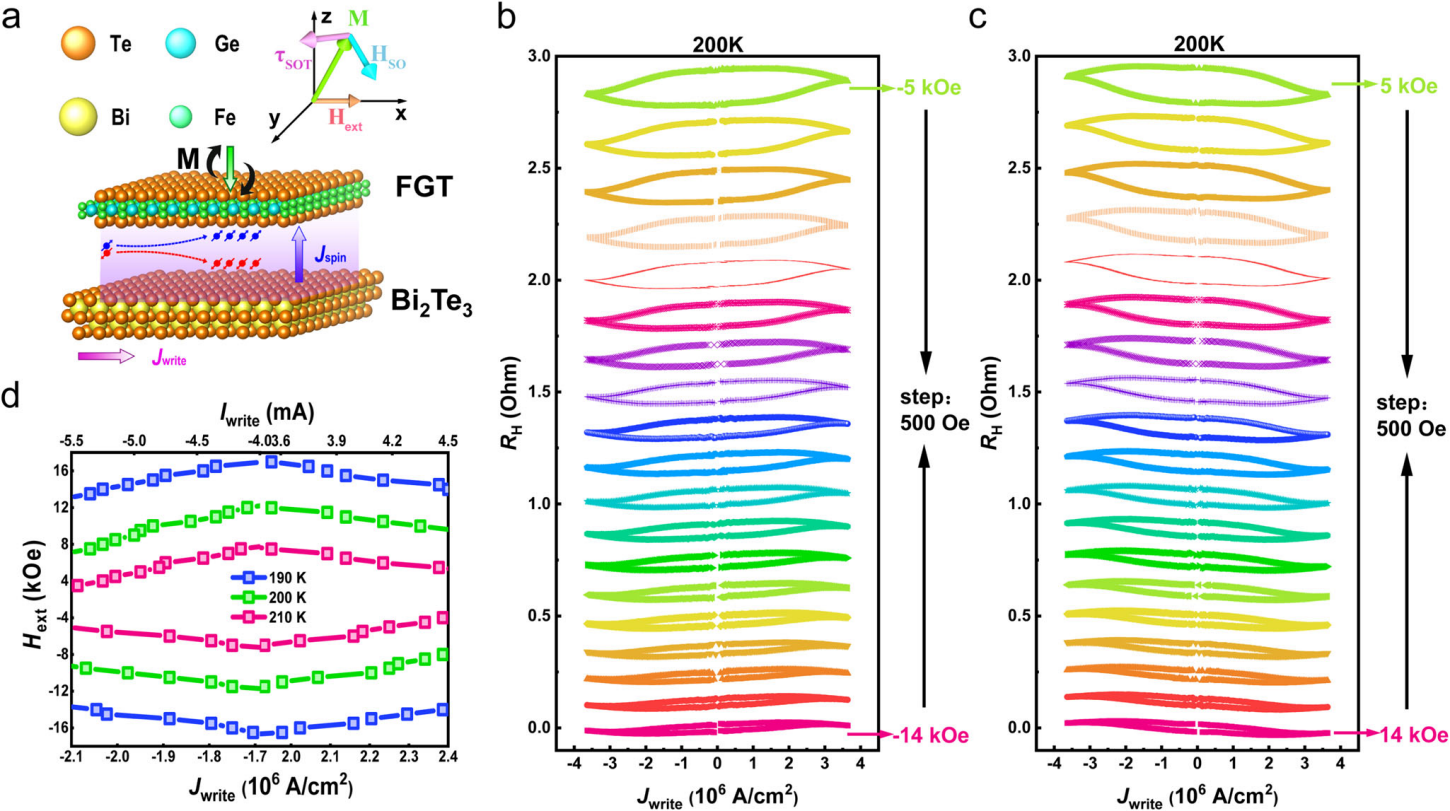
Spin–orbit torque-induced magnetization switching behaviors in Bi_2_Te_3_(8)/Fe_3_GeTe_2_(3) heterostructure. **a** Geometric structure diagram of SOT switching in Bi_2_Te_3_/FGT heterostructure. The effective spin–orbit field (*H*_so_) exerts a spin torque (*τ*_SOT_) for magnetization switching. **b**, **c** Current-induced magnetic switching at 200 K with different in-plane magnetic fields, showing the opposite SOT switching chirality when reversing the magnetic field. **d** Phase diagram for SOT switching in different writing currents and in-plane magnetic fields at 190 K, 200 K, and 210 K.

Here, Bi_2_Te_3_(8)/FGT(3) was taken as the research object for SOT switching. Figure 2a shows the geometric diagram of SOT-driven magnetic switching dynamics in the vdW heterostructure of Bi_2_Te_3_ and FGT. By injecting sufficient spin current density (*J*_spin_) from Bi_2_Te_3_, SOT enables the magnetization (M) switching in the adjacent ferromagnetic layer above the critical writing current density (*J*_write_). It is worth noting that the injected *J*_write_ is orthogonal to the accumulated spin polarization direction and the generated *J*_spin_ direction. Here, *J*_write_ can be determined as ${J}_{{{{{\rm{write}}}}}}={I}_{{{{{\rm{write}}}}}}/\left[w*\left({t}_{{{{{\rm{{Bi}}}}}}_{2}{{Te}}_{3}}+{t}_{{{{{\rm{FGT}}}}}}\right)\right]$, where *w* = 30 μm is the width of the Hall-bar^25^. The effective spin–orbit field (*H*_so_) induced by the spin current is along the tangential of M and could tilt M up or down to get the positive or negative z component (*M*_*z*_). Usually, for PMA samples, an additional external magnetic field (*H*_ext_) needs to be applied during the measurement process to break the mirror symmetry for the deterministic SOT switching^48^. Thus, when sweeping the applied charge current with an external in-plane field, the SOT from the charge-spin conversion in Bi_2_Te_3_ would induce the magnetization reversal in the ferromagnetic layer.

To demonstrate the SOT switching in the Bi_2_Te_3_/FGT heterostructure, a series of in-plane magnetic fields were applied with a 10-ms pulse current along the Hall bar to obtain a deterministic switch polarity. We observe that the M changes steadily as the applied *J*_write_ increases, and a complete magnetic switching is achieved when the *J*_write_ reaches approximately 4 × 10^6^ A/cm^2^ at 200 K, as shown in Fig. 2b. When the current density is greater than 2.5 ×10^6^ A/cm^2^, Hall-resistance (*R*_H_) begins to decrease after reaching the maximum, which is related to a Joule heating effect. Our explanation for this case is that the magnetic interactions of FGT are not sufficient to fight against the thermal fluctuations, resulting in a decrease of M^46^. Interestingly, the critical *J*_write_ for SOT switching is much smaller than the values reported in FGT/Pt heterostructure, probably due to the high efficiency of charge-spin conversion in TI non-trivial origin. As the applied magnetic field is reversed, the opposite chirality of the SOT switching curve demonstrates the typical characteristics of SOT in the PMA sample, as shown in Fig. 2c. Moreover, the device was measured at different temperatures of 210 K and 190 K. We found that as the temperature decreases, the range of the applied magnetic field to achieve the SOT switch gradually increases (Supplementary Fig. S2 and Fig. S3). For an in-depth understanding of the switching behavior, we summarize the dependence of the current density on the applied magnetic field for SOT switching at different temperatures in the phase diagram of Fig. 2d. Here, the critical switching current density (*J*_sw_) that is defined as the sign change in *R*_H_ is gradually reduced at the higher magnetic field. The deterministic switching happens in the large field and current region, while both up and down magnetization states are possible in the intermediate region associated with a small field and current region. Additionally, the switching current decreases with increasing temperature, which is attributed to the simultaneous decrease in *M*_s_, as already proved in Fig. 1f.

### Harmonic Hall measurements in Bi_2_Te_3_/FGT heterostructure

To quantitatively evaluate the SOT efficiency, we use the harmonic Hall measurement to characterize the effective field of SOT, which could provide a solid understanding of each SOT component, as well as its influencing factors. We apply a small sinusoidal current (*J*_a.c._) to the channel of the device and then generate a SOT in the ferromagnetic layer, which will be decomposed into two mutually orthogonal vector components: damping-like torque ${\tau }_{{{{{\rm{DL}}}}}} \sim m\times \left(\sigma \times m\right)$ and field-like torque ${\tau }_{{{{{\rm{FL}}}}}} \sim \sigma \times m$^49^. In the measurement, the frequency is fixed at 133.33 H_*z*_ through the lock-in amplification, and the magnetization oscillation of M around the equilibrium position generates the harmonic Hall signals, including the in-phase first harmonic Hall voltage (*V*_1ω_) and out-of-phase second harmonic Hall voltage (*V*_2*ω*_). We analyzed the second-harmonic anomalous Hall resistance $({R}_{{{{{\rm{AHE}}}}}}^{2{{{{{\rm{\omega }}}}}}})$ and planar Hall resistance $({R}_{{{{{\rm{PHE}}}}}}^{2{{\omega }}})$ to determine the current-induced SOT effective field. After applying an external magnetic field (*H*_*x*_) to the *x*-axis, the second harmonic Hall resistance ${R}_{{xy}}^{2{{{{{\rm{\omega }}}}}}}$ could be obtained by the following equation for values of *H*_*x*_ larger than the magnetic anisotropy field *H*_k_:^27^

1 $${R}_{{xy}}^{2{{{{{\rm{\omega }}}}}}}\,=	\,{R}_{{{{{\rm{AHE}}}}}}^{2{{{{{\rm{\omega }}}}}}}+{R}_{{{{{\rm{PHE}}}}}}^{2{{{{{\rm{\omega }}}}}}}+{R}_{{{{{\rm{ANE}}}}}}\frac{{H}_{x}}{\left|{H}_{x}\right|}+{R}_{{{{{\rm{offset}}}}}}\\=	\, \frac{{R}_{A}}{2}\frac{{H}_{{{{{\rm{DL}}}}}}}{\left|{H}_{x}\right|-{H}_{k}}+{R}_{p}\frac{{H}_{{{{{\rm{FL}}}}}}}{\left|{H}_{x}\right|}+{R}_{{{{{\rm{ANE}}}}}}\frac{{H}_{x}}{\left|{H}_{x}\right|}+{R}_{{{{{\rm{offset}}}}}}$$

where *H*_DL_ and *H*_FL_ are the damping-like effective field ($\propto m\times \sigma$) and field-like effective field ($\propto \sigma$), respectively. *R*_p_ and *R*_A_ are the planar Hall resistance and anomalous Hall resistance, respectively. *R*_offset_ is the resistance offset. *R*_ANE_ is the transverse resistance contributed by the anomalous Nernst effect and other spin-related thermoelectric effects^50^. For the damping-like effective term, it decreases as the external field increases. For the thermal-related term, its sign changes as the external field direction reverse, while its magnitude keeps constant. Usually, the *R*_p_ is extremely small compared to the anomalous Hall counterpart and thus ${R}_{{xy}}^{2{{{{{\rm{\omega }}}}}}}$ mainly originates from the damping-like effective field term and thermal-effect term. Figure 3a displays a series of ${R}_{{xy}}^{2{{{{{\rm{\omega }}}}}}}-{H}_{x}$ curves under different applied *J*_a.c._ at 200 K. It demonstrates a distinct field dependence, while a step function could also be observed, which means that in addition to the contribution of the damping-like Hall signal, it also has a thermal contribution in our sample. With increasing the *J*_a.c._, both signals are enhanced. The inset in Fig. 3a schematically illustrates the second harmonic Hall signal that comes from the SOT-induced magnetization oscillation around the equilibrium position. For quantitatively characterizing the thermal signal, we carried out the temperature-dependent ${R}_{{xy}}^{2{{{{{\rm{\omega }}}}}}}-{H}_{x}$ at a fixed *J*_a.c._ to provide further evidence. Here, we defined ${R}_{{{{{\rm{ANE}}}}}}=({R}_{{xy}({{{{\rm{sat}}}}}\_{max} )}-{R}_{{xy}({{{{\rm{sat}}}}}\_{min} )})/2$ to express the thermal contribution, where ${R}_{{xy}({{{{\rm{sat}}}}}\_{{{{{\mathrm{max}}}}}} )}$ and ${R}_{{xy}({{{{{\rm{sat}}}}}}\_{{{{{\rm{min}}}}}} )}$ are defined as the maximum and minimum values of second-harmonic Hall resistance under a saturated magnetic field^27^. As temperature decreases, the *R*_ANE_ becomes much larger, which implies thermal contribution is more pronounced at low temperatures. To understand the origin of the thermal-related effect, it is worth noting that the metallic and topological nature of FGT could cause a large anomalous Nernst effect (ANE)^43^. In our sample, the top layer above FGT is air with ambient temperature, while the bottom layer is Bi_2_Te_3_ with a large current. The vertical thermal gradient from the asymmetric structure may contribute to the thermal current, thus inducing the ANE. Nevertheless, we could differentiate the ANE and the SOT-induced second-harmonic Hall resistance through their magnetic field dependence. Figure 3b displays the influence of the FGT’s large ANE on the heterostructure, and the left inset is a schematic diagram of the step function of the thermal contribution^51^.

**Fig. 3:**
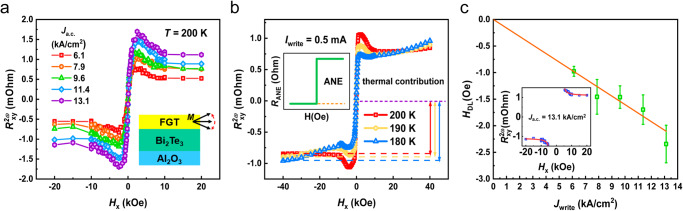
Harmonic measurements under different temperatures in Bi_2_Te_3_(8)/Fe_3_GeTe_2_(3) heterostructure. **a** Second-harmonic Hall resistance (${R}_{{xy}}^{2{{{{{\rm{\omega }}}}}}}$) under different *J*_a.c._ at 200 K. The inset displays the oscillation of the magnetic moment at the equilibrium position in harmonic measurement. **b** Second-harmonic Hall resistance (${R}_{{xy}}^{2{{{{{\rm{\omega }}}}}}}$) as a function of in-plane magnetic field (*H*_x_) at different temperatures under a constant write current density. The inset schematically displays the field dependencies of anomalous Nernst resistance. **c** Damping-like effective field (*H*_DL_) as a function of the current density extracted by fitting the second harmonic Hall signal. The inset shows a typical ${R}_{{xy}}^{2{{{{{\rm{\omega }}}}}}}$–*H*_x_ curve under the large magnetic field range for fitting out the *H*_DL_. The error bars denote the standard deviation of multiple measurements.

Relying on the above analysis, we extract *H*_DL_ and display the dependence of *H*_DL_ on corresponding *J*_a.c._ at 200 K, as shown in Fig. 3c. The resistivities of the Bi_2_Te_3_ and FGT layers of different thicknesses are evaluated (Supplementary Fig. S4). By fitting the process in the large in-plane magnetization region with the formula (1), ${H}_{{DL}}/{J}_{{write}}$ is ~160.2 Oe per MA/cm2 in the inset of Fig. 3c. The SOT efficiency (*ξ*_DL_) can be obtained using^52^,

2 $${\xi }_{{{{{\rm{DL}}}}}}=\frac{2e{M}_{{{{{{\rm{s}}}}}}}t}{{{\hslash }}}\frac{{H}_{{{{{{\rm{DL}}}}}}}}{{J}_{{{{{{\rm{a}}}}}}.{{{{{\rm{c}}}}}}.}}$$

where *e* and ℏ are the electron charge and reduced Plank constant, respectively, *t* represents the ferromagnetic layer thickness. Accordingly, the value of the *ξ*_DL_ is determined to be ~5.3 in Bi_2_Te_3_(8)/FGT(3) structure at 200 K.

**Fig. 4:**
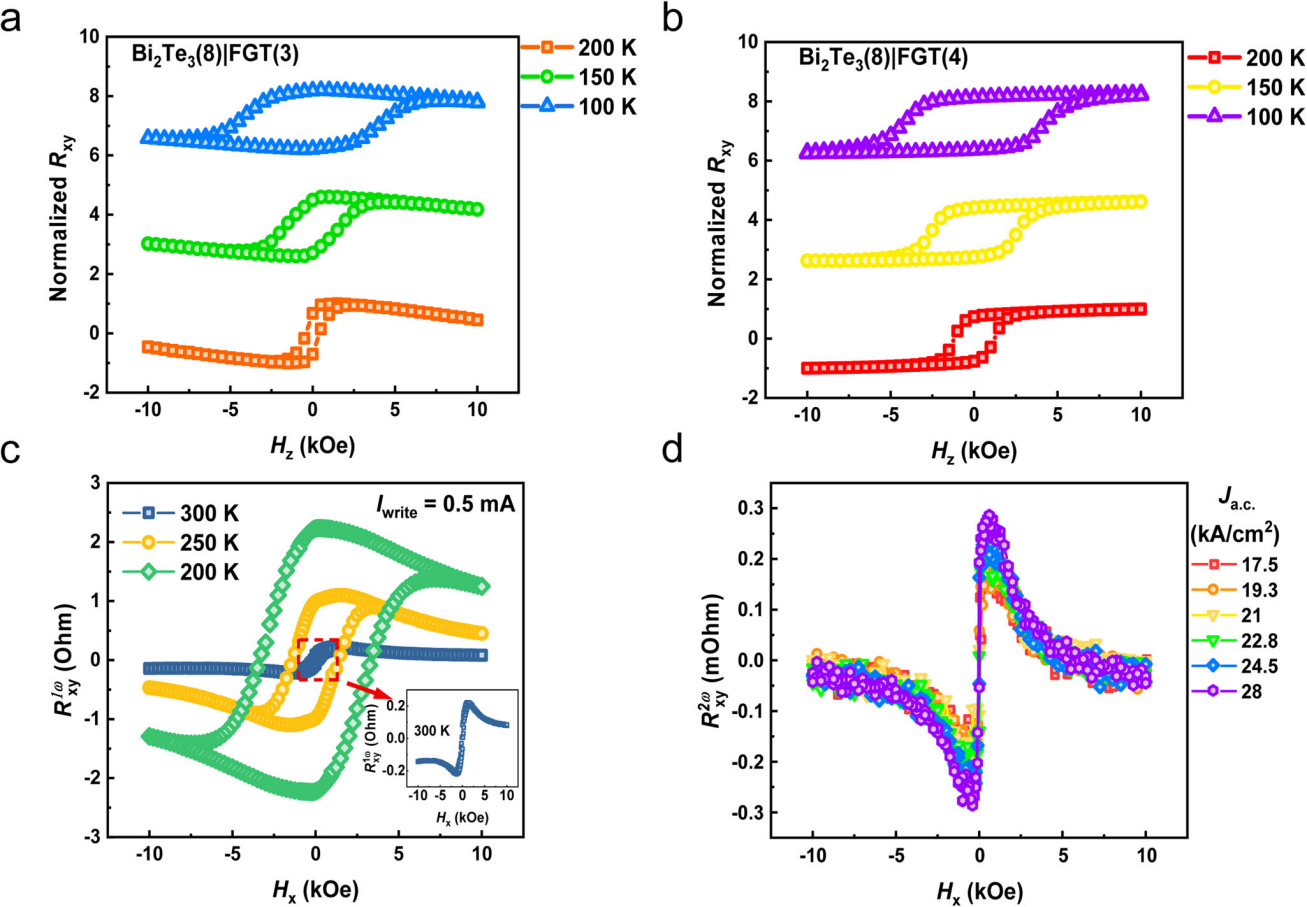
Comparative out-of-plane field anomalous Hall results in Bi_2_Te_3_/Fe_3_GeTe_2_ heterostructures and in-plane field harmonic Hall signals in Bi_2_Te_3_(8)/Fe_3_GeTe_2_(4) heterostructure. **a**, **b** Normalized anomalous Hall resistance (*R*_xy_) as a function of the out-of-plane external magnetic field (*H*_z_) at 100 K, 150 K, and 200 K in Bi_2_Te_3_(8)/Fe_3_GeTe_2_(3) and Bi_2_Te_3_(8)/Fe_3_GeTe_2_(4) heterostructures, respectively. **c** First-harmonic Hall resistance as a function of the in-plane external field under different temperatures in Bi_2_Te_3_(8)/Fe_3_GeTe_2_(4) heterostructure. **d** Second-harmonic Hall resistance under different applied *J*_a.c._ at room temperature, showing the SOT enhancement with increasing current.

To eliminate the thermal contribution caused by the ANE of FGT on the SOT efficiency, we adjusted the thickness of FGT to manipulate the shunting current in the Bi_2_Te_3_ for lowering the thermal gradient in the Bi_2_Te_3_/FGT heterostructure^53^. Moreover, we conduct the measurements with different *I*_*dc*_ while sweeping *H*_*x*_ to observe the variation of *R*_*xy*_, and find that the DC of 0.5 mA to 1.5 mA has no significant effect on the heterostructure, which further verifies that this thickness of the heterostructure has better thermal stability (Supplementary Fig. S5). Figures 4a, b displays the out-of-plane external magnetic field-dependent *R*_*xy*_ on Bi_2_Te_3_/FGT heterostructures with varying thicknesses of FGT (3 nm and 4 nm) at 100 K, 150 K, and 200 K. We normalize its *R*_*xy*_ to facilitate comparison. It is worth noting that the *R*_*xy*_ of Bi_2_Te_3_(8)/FGT(3) has an obviously negative ordinary Hall slope in the saturated magnetic field region, which is similar to that from the Bi_2_Te_3_ Hall signal, indicating that Bi_2_Te_3_ in the heterostructure has a large shunting effect. In contrast, the *R*_xy_ of Bi_2_Te_3_(8)/FGT(4) shows only the anomalous Hall signal from FGT, which well proves the shunting effect in Bi_2_Te_3_ has been significantly reduced due to more conducting in FGT after increased thickness. The PMA feature was further verified by performing first-harmonic Hall measurement with an in-plane magnetic field, and the results under different temperatures are shown in Fig. 4c. Subsequently, we conducted the second-harmonic Hall measurements and displayed *R*_2*ω*_ signals as a function of *H*_*x*_ under different *J*_write_ in Fig. 4d. Interestingly, the step function arising from ANE disappears, which well matches our above prediction. Followed by Eqs. (1) and (2), the room temperature *ξ*_DL_ is estimated to be ~0.7, which indicates the strong SOC characteristics of TI at room temperature.

To verify the conjecture and understand the related mechanism in our sample, we give a systematic discussion about the temperature dependence of *ξ*_DL_. Unlike traditional heavy metals, TI exhibits a topologically-protected non-trivial surface state, which is composed of a single massless Dirac fermion with two spin-splitting bands on the surface. When the time-reversal symmetry is broken, the surface state will open a gap. The bulk Hamiltonian projected onto the surface state is described as^54,55^

3 $${H}_{{{{{\rm{surf}}}}}}\left({\overrightarrow{{k}_{x}}},\,{\overrightarrow{k}_{y}}\right)=\upsilon {{\hslash }}\left({\overrightarrow{{\sigma }^{x}}\,\overrightarrow{{k}_{y}}}-{\overrightarrow{{\sigma }^{y}}\,\overrightarrow{{k}_{x}}}\right)$$

where $\upsilon$ is the velocity of the surface state and $k$ is the Dirac electron momentum. When the *J*_write_ is applied to TI, the spin of the Dirac electron is locked, and the movement of the Fermi surface in the *k*-space will produce controllable spin polarization. Another important origin of SOT is the spin Hall effect (SHE) of the bulk state, which utilizes the bulk SOC in TI to convert non-polarized write current into the spin current. Due to the asymmetric scattering of conductive electrons, the spin-up and spin-down are deflected in opposite directions, forming a transverse spin current.

**Fig. 5:**
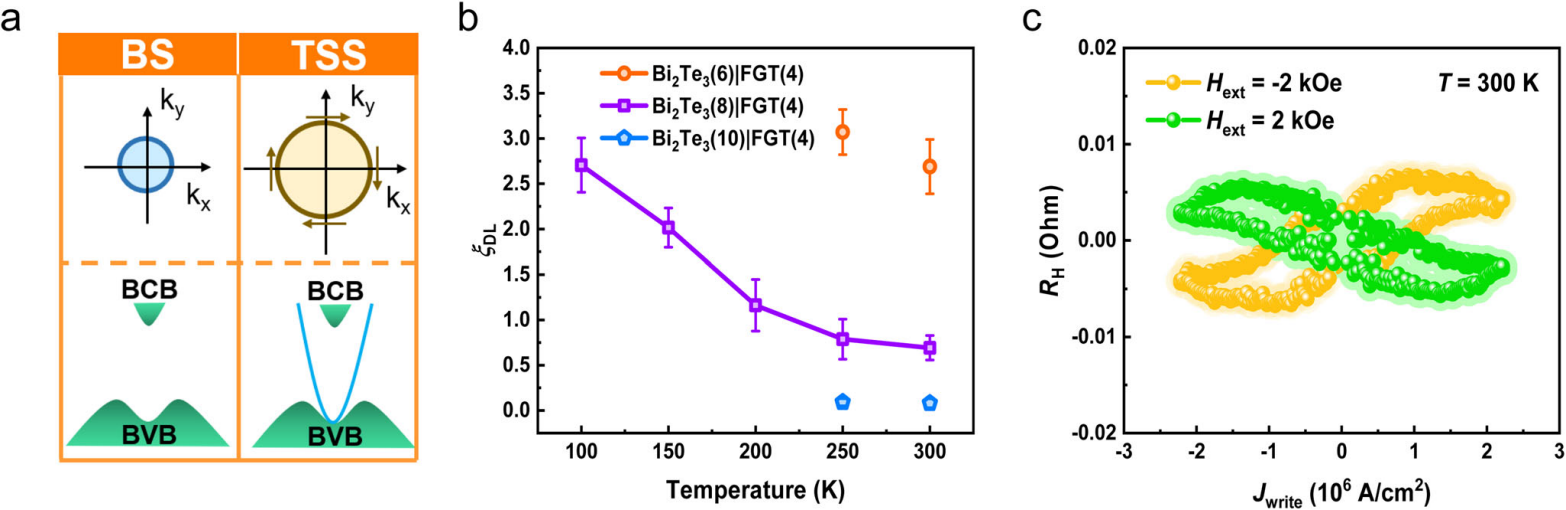
SOT efficiency characterization and current-induced room-temperature switching in Bi_2_Te_3_/Fe_3_GeTe_2_(4) heterostructures. **a** Illustration of the concept of charge-spin conversion via bulk state and topological surface states. **b** The SOT efficiency (*ξ*_DL_) at different temperatures and thicknesses, showing the enhanced SOT switching from the topological surface state. The error bars denote the standard deviation of multiple measurements. **c** Current-induced magnetization switching under ±2 kOe at room temperature.

Figure 5a displays the schematic spin-related band structure of the TSS and bulk state. Both of them coexist in the film^56^, and either the surface or bulk state would provide a contribution to the final SOT. To gain insights into how large surface contribution to the SOT, temperature-dependent SOT efficiency and its relation to the *E*_F_ were carried out for analysis. Figure 5b shows the precise SOT efficiency results through harmonic Hall measurements in the Bi_2_Te_3_(8)/FGT(4) heterostructure. Here, we examined the accuracy of different fitting methods on the calculation results from the perspective of the extended Landau–Lifshitz–Gilbert equation and anisotropy (more details in Supplementary Fig. S6). We found that *ξ*_DL_ exhibited a drastic nonlinear growth with a decrease in temperature^57^. At room temperature, the *E*_F_ predominantly resides within the highly conductive bulk state, but as the temperature decreases, it shifts downwards toward the Dirac cone with a reduced bulk state (Supplementary Fig. S7). The fact that TI with reduced bulk conductance leads to a higher SOT efficiency suggests that the TSS renders significant contributions to the efficient SOT. Furthermore, additional heterostructures with different TI thicknesses (Bi_2_Te_3_(6)/FGT(4) and Bi_2_Te_3_(10)/FGT(4)) were prepared for comparison with previous samples. The SOT efficiency from the harmonic measurements has undergone a dramatic increase to ~2.69, which further proves the substantial surface contribution of Bi_2_Te_3_ at room temperature (Supplementary Fig. S8). Besides, it is worth noting that the Rashba spin-splitting surface state in the two-dimensional electron gas (2DEG) may coexist with the TSS in Bi_2_Te_3_ due to the band bending and structural inversion asymmetry^5,57^. However, the Rashba effective field is expected to increase gradually as the temperature rises in the semiconductor system^58,59^, different from our experimental results^60^. Hence, we conclude that the Rashba-split surface state is not the primary physical mechanism for SOT switching^26^.

For the chirality of SOT, the relationship between the *J*_spin_ and the *J*_write_ can be expressed by the following formula:^61^

4 $${J}_{{{{{\rm{spin}}}}}}=\frac{{{\hslash }}}{2e}{\theta }_{{{{{\rm{SH}}}}}}\left({J}_{{{{{\rm{write}}}}}}\times \vec{\sigma }\right)$$

where *θ*_SH_ is the spin Hall angle, *σ* is the polarization of the spin, and its direction is orthogonal to the direction of the *J*_write_. For non-ferromagnetic materials that provide spin currents, the spin direction of the top surface and the bottom surface is opposite, and its chirality is defined by the sign of *θ*_SH_. Compared with our results, the SOT switching in FGT/Pt heterostructures shows the same chirality, further accurately confirming our conclusion^45,46^. As reported previously, the chirality from TSS is the same as that from the bulk state with positive spin Hall angle^27,62^. Finally, the SOT switching of the FGT layer was successfully demonstrated in the Bi_2_Te_3_(8)/FGT(4) heterostructure at room temperature when 10-ms pulse currents were applied to the Hall bar with an ${H}_{{{{{\rm{ext}}}}}}=\pm 2{{{{{\rm{kOe}}}}}}$ under several consecutive sweeps, as shown in Fig. 5c. It sets a new stage for exploring all-vdW SOT devices.

For clarity, we summarize the switching write current density, SOT efficiency, and its realized maximum temperature of several representative heterostructures for comprehensively understanding the SOT feature in the Bi_2_Te_3_/FGT heterostructure, and the results are presented in Table 1. The heavy metal Pt is generally used as the preferred material to achieve SOT switching of FGT at low temperatures. It is worth noting that the minimum *ξ*_DL_ in the FGT/Pt heterostructure reported by Alghamdi et al. is as large as the maximum of the CoFeB/Pt structure^45^, demonstrating the vdW FGT superiority. In comparison with our sample, the large *ξ*_DL_ value well proves the TI of Bi_2_Te_3_ is superior for charge-spin conversion with 2D vdW ferromagnet. Compared to previously reported Bi_2_Te_3_-based heterostructures, our sample also has a significant advantage in SOT efficiency. Recently, WTe_2_/FGT heterostructures have been found to achieve SOT properties and relatively excellent performance, but still at low temperature^63,64^. These results obtained with the same characterization method may provide evidence that the interfacial spin transparency could be significantly enhanced by the vdW-gapped interface between Bi_2_Te_3_ and FGT due to the optimized growth method. In such a case, the *ξ*_DL_ is related to the internal *θ*_SH_ of TI and the interfacial spin transparency *T*_int_^29,46^. The interfacial spin transparency could be mainly determined by the mechanisms of spin backflow and spin memory loss, which could be characterized by the effective spin-mixing conductance and the spin conductance of the non-ferromagnetic layer^29^. A good interface contributes to the transparency during spin transport at room temperature, which is one of the most important factors in achieving energy-efficient SOT switching in an all-vdW heterostructure and highlights the strong SOC characteristics of TI.

**Table 1:** SOT characteristics in several typical heterostructures of 2D vdW ferromagnets

	Maximum temperature of SOT switching	Switching write current density	SOT efficiency
Pt/Fe_3_GeTe_2_ (ref. ^45^.)	180 K	~2.5 × 10^7^ A/cm^2^	0.14
Pt/Fe_3_GeTe_2_ (ref. ^46^)	120 K	~7.4 × 10^6^ A/cm^2^	0.12
WTe_2_/Fe_3_GeTe_2_ (ref. ^60^)	135 K	~6.5 × 10^6^ A/cm^2^	/
WTe_2_/Fe_3_GeTe_2_ (ref. ^63^)	190 K	~4.2 × 10^6^ A/cm^2^	/
WTe_2_/Fe_3_GeTe_2_ (ref. ^64^)	160 K	~3.5 × 10^6^ A/cm^2^	4.6
Bi_2_Te_3_/Fe_3_GeTe_2_ (this work)	300 K	~4 × 10^6^ A/cm^2^ (200 K); ~2.2 × 10^6^ A/cm^2^ (300 K)	0.7 (300 K)

To summarize, the wafer-scale vdW Bi_2_Te_3_/FGT heterostructure prepared by MBE has successfully realized room-temperature ferromagnetism and current-driven SOT switching. We employed the harmonic Hall signals to accurately estimate the SOT efficiency, which was as high as ~0.7 at room temperature, and this value could be further increased to ~2.69 with decreasing TI thickness. Together with the temperature-dependent measurement, the high charge-spin conversion efficiency is mainly attributed to the improved interfacial spin transparency and nontrivial topological origin of the all-vdW Bi_2_Te_3_/FGT heterostructure. The realization of room-temperature ferromagnetism and SOT switching together in Bi_2_Te_3_/FGT heterostructure establishes a promising route for the development of all-vdW heterostructures and lays the foundation for implementation of room-temperature 2D vdW spintronic devices in the future.

## Methods

### Sample growth

The (0001) sapphire substrate was used to grow the sample. High-purity Bi, Fe, Ge, and Te were evaporated from Knudsen effusion cells in the MBE system with a base vacuum of 10^−10^ Torr. After degassing at high temperature, the substrate was cooled down to 300 °C for growing both the FGT thin film and Bi_2_Te_3_/FGT heterostructure with a growth rate of ~0.05 Å/s, and the sample quality was monitored by an in situ RHEED system.

### Characterization

The morphologies of the samples were investigated by AFM. The microstructure and composition were comprehensively characterized by XRD and HAADF in STEM mode. The cross-section TEM sample was prepared by a focused ion beam. MOKE and SQUID were employed to measure their magnetic properties. Furthermore, the magnetotransport studies were carried out in the Quantum Design physical property measurement system.

### Supplementary information

Supplementary information

## Data Availability

All data generated in this study are provided in the paper and Supplementary Information/Source Data file. Additional data related to this study are available from the corresponding author upon reasonable request.
