# Supplementary material for: Room temperature energy-efficient spin-orbit torque switching in two-dimensional van der Waals Fe3GeTe2 induced by topological insulators
Source: Nat Commun. 2023 Aug 24;14:5173. doi: 10.1038/s41467-023-40714-y (PMC10449904; doi:10.1038/s41467-023-40714-y)
Supplement: Supplementary file 1 — Supplementary information [file 41467_2023_40714_MOESM1_ESM.pdf]

Room temperature energy-efficient spin-orbit torque switching in two-dimensional van der Waals Fe_3GeTe_2 induced by topological insulators

Haiyu Wang[1,2,7], Hao Wu[3,7], Jie Zhang[1,7], Yingjie Liu[1], Dongdong Chen[4], Chandan Pandey[1], Jialiang Yin[1], Dahai Wei[4], Na Lei[1], Shuyuan Shi[1], Haichang Lu[1], Peng Li[5], Albert Fert[1,6], Kang L. Wang[3], Tianxiao Nie[1*], Weisheng Zhao[1*]

[1]Fert Beijing Institute, MIIT Key Laboratory of Spintronics, School of Integrated Circuit Science and Engineering, Beihang University, Beijing, China.
[2]Shenyuan Honors College, Beihang University, Beijing, China.
[3]Department of Electrical and Computer Engineering, and Department of Physics and Astronomy, University of California, Los Angeles, California, USA.
[4]State Key Laboratory of Superlattices and Microstructures, Institute of Semiconductors, Chinese Academy of Sciences, P.O. Box 912, Beijing, China.
[5]Department of Electrical and Computer Engineering, Auburn University, Alabama, USA.
[6]Unité Mixte de Physique, CNRS, Thales, Univ. Paris-Sud, University of Paris-Saclay, Palaiseau, France.

[7]These authors contributed equally: Haiyu Wang, Hao Wu, Jie Zhang.
[*]Corresponding authors. E-mail: nietianxiao@buaa.edu.cn; wszhao@buaa.edu.cn

Supplementary information

Contents:

1. Structural characteristics of FGT, Bi_2Te_3, and Bi_2Te_3/FGT heterostructures

2. SOT-induced magnetic switching in Bi_2Te_3/FGT(3) heterostructures at 210 K

3. SOT-induced magnetic switching in Bi_2Te_3/FGT(3) heterostructures at 190 K

4. Temperature-dependent resistivity from Bi_2Te_3 and FGT thin films

5. Magnetotransport properties of Bi_2Te_3/FGT heterostructures

6. Harmonic Hall voltage measurement and SOT efficiency calculation in Bi_2Te_3(8)/FGT(4)

7. Estimation of Fermi level in Bi_2Te_3(8) structure at different temperatures

8. Harmonic Hall voltage measurements in Bi_2Te_3(6)/FGT(4) and Bi_2Te_3(10)/FGT(4) heterostructures at 300K

1. Structural characteristics and magnetic property of FGT, Bi_2Te_3, and Bi_2Te_3/FGT heterostructures

To understand the microstructure and surface morphology, the structural characteristics of FGT, Bi_2Te_3, and Bi_2Te_3/FGT heterostructure were measured, and the results are shown in Fig. S1. The atomic force microscopy (AFM) results corresponding to FGT and Bi_2Te_3 are shown in Fig. S1a and Fig. S1b, respectively. Bi_2Te_3 shows a terraced surface that is well consistent with the previously-reported surface morphology of Bi_2Te_3[1,2]. In contrast, the FGT shows a smoother surface rather than a terrace. The crystal structures were characterized by X-ray diffraction (XRD), and typical results from pure FGT and Bi_2Te_3 are respectively shown in Fig. S1c and Fig. S1d. According to PDF Card #75-5620 and #15-0863, the XRD peaks can be determined to be a series of {002} planes from FGT and {003} planes from Bi_2Te_3, respectively. The XRD scanning result of Bi_2Te_3/FGT heterostructure is shown in Fig. S1e, in which the corresponding peaks from FGT and Bi_2Te_3 are marked separately in red and blue, showing the high-quality single crystallinity of FGT and Bi_2Te_3 in Bi_2Te_3/FGT heterostructure. Typical RHEED results are shown in Fig. S1f, which clearly demonstrates two types of steaky patterns in different crystal orientations, corresponding to [100] and [120] incident beams respectively. Therefore, we confirm that the high-quality crystal was obtained with no in-plane domains. Meanwhile, anomalous Hall measurement was carried out to characterize their magnetic properties, and typical results from FGT and Bi_2Te_3/FGT are shown in Fig. S1g-i, which clearly demonstrate the PMA features.

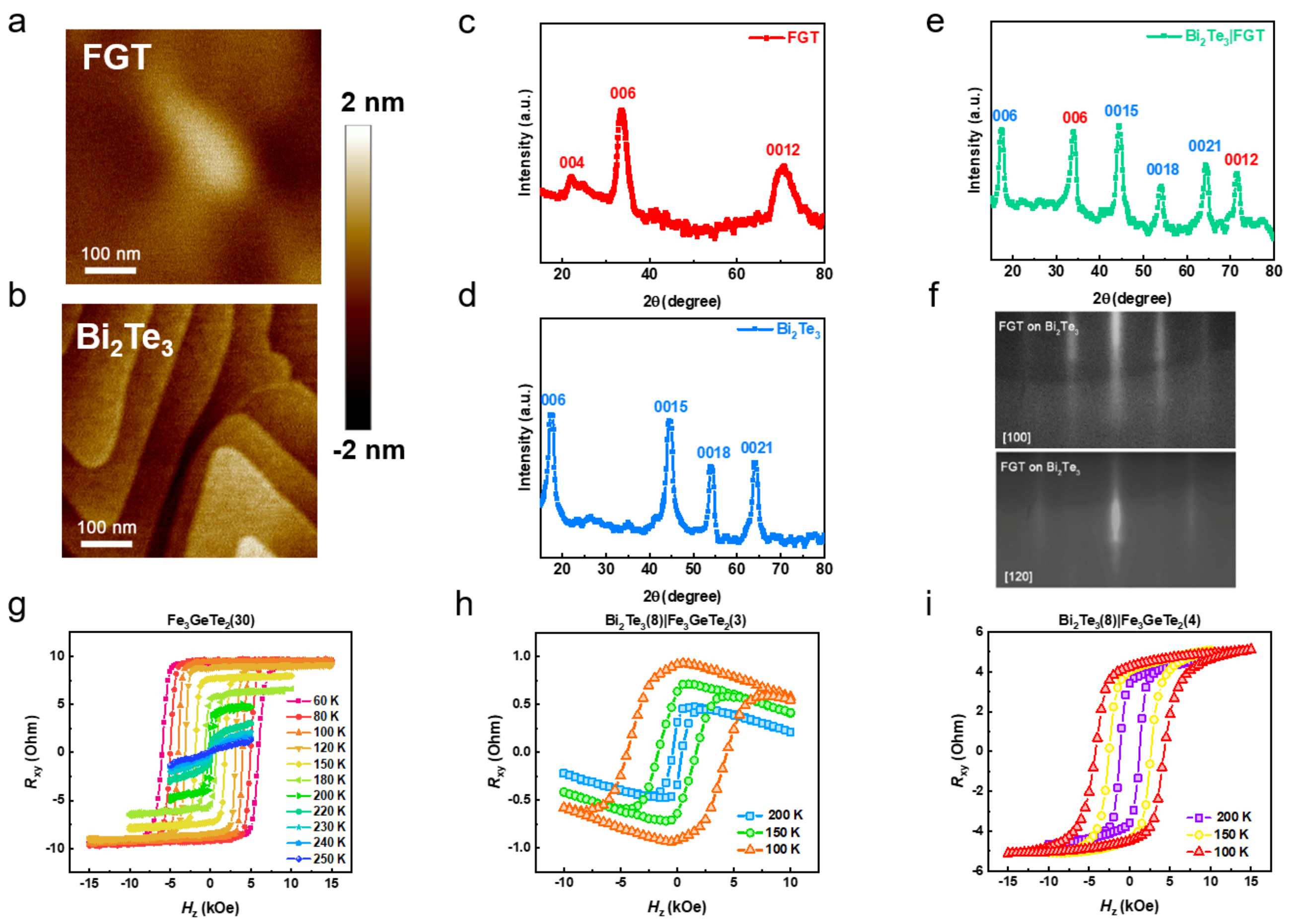

Figure S1 | Structural characteristics and magnetic property of FGT, Bi_2Te_3, and Bi_2Te_3/FGT heterostructures. a, b AFM images of FGT and Bi_2Te_3, respectively showing the smooth surface of FGT and terrace surface of Bi_2Te_3. **c-e** XRD measurements of pure FGT, pure Bi_2Te_3, and Bi_2Te_3/FGT films show the high-quality single crystallinity. **f** RHEED images of Bi_2Te_3/FGT heterostructure, further showing the high-crystalline quality. **g-i** Anomalous Hall resistance of 30nm FGT, Bi_2Te_3(8)|FGT(3) and Bi_2Te_3(8)|FGT(4) at different temperatures, respectively.

2. SOT-induced magnetic switching in Bi_2Te_3/FGT(3) heterostructure at 210 K

In addition to the current-induced magnetic switching in the Bi_2Te_3/FGT(3) heterostructure at 200 K in the main text, we carried out the identical SOT measurement at another temperature of 210 K. Similarly, when applying a constant external in-plane field with sweeping the applied J_{write}, the SOT from the charge-spin conversion in Bi_2Te_3 can induce the magnetization switching of the FGT layer.

As the external magnetic field increases, the switching current density decreases. When the applied magnetic field is reversed, the SOT switching shows an opposite chirality. All these results well demonstrate the magnetization switching behaviors in the existence of SOT.

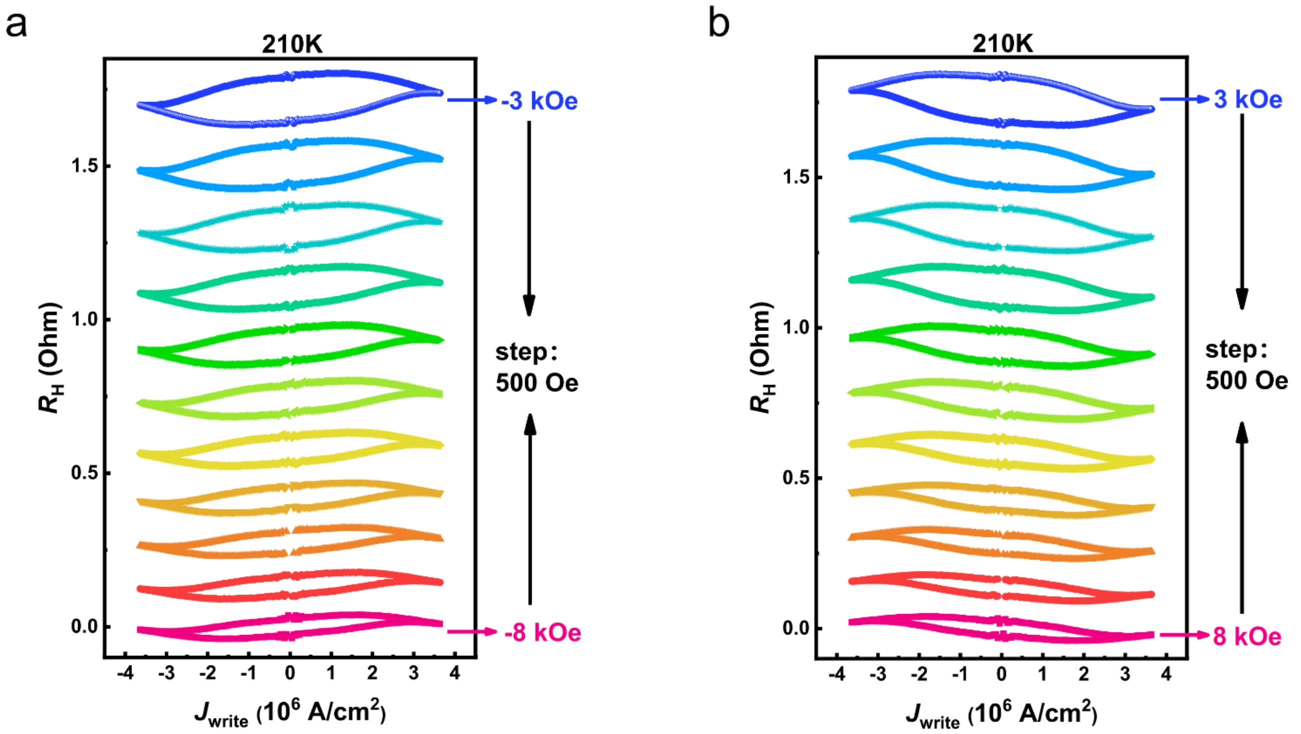

Figure S2 | Spin-orbit torque induced magnetization switching behaviors in Bi_2Te_3(8)/FGT(3) at 210 K. a, b SOT-induced magnetization switching behaviors at 210 K under different applied in-plane magnetic field, which show the field-dependent switching chirality and critical switching current density.

3. SOT-induced magnetic switching in Bi_2Te_3/FGT(3) heterostructures at 190 K

Utilizing the identical SOT measurement, we performed the current-driven magnetic switching on Bi_2Te_3(8)/FGT(3) at 190 K, and typical results are shown in Fig. S3. The switching chirality is the same as other temperatures when reversing the external in-plane magnetic field. Based on the above switching behavior, we summarize the temperature-dependent phase diagram of magnetic states in the presence of external magnetic field and current density, as already shown in the main text.

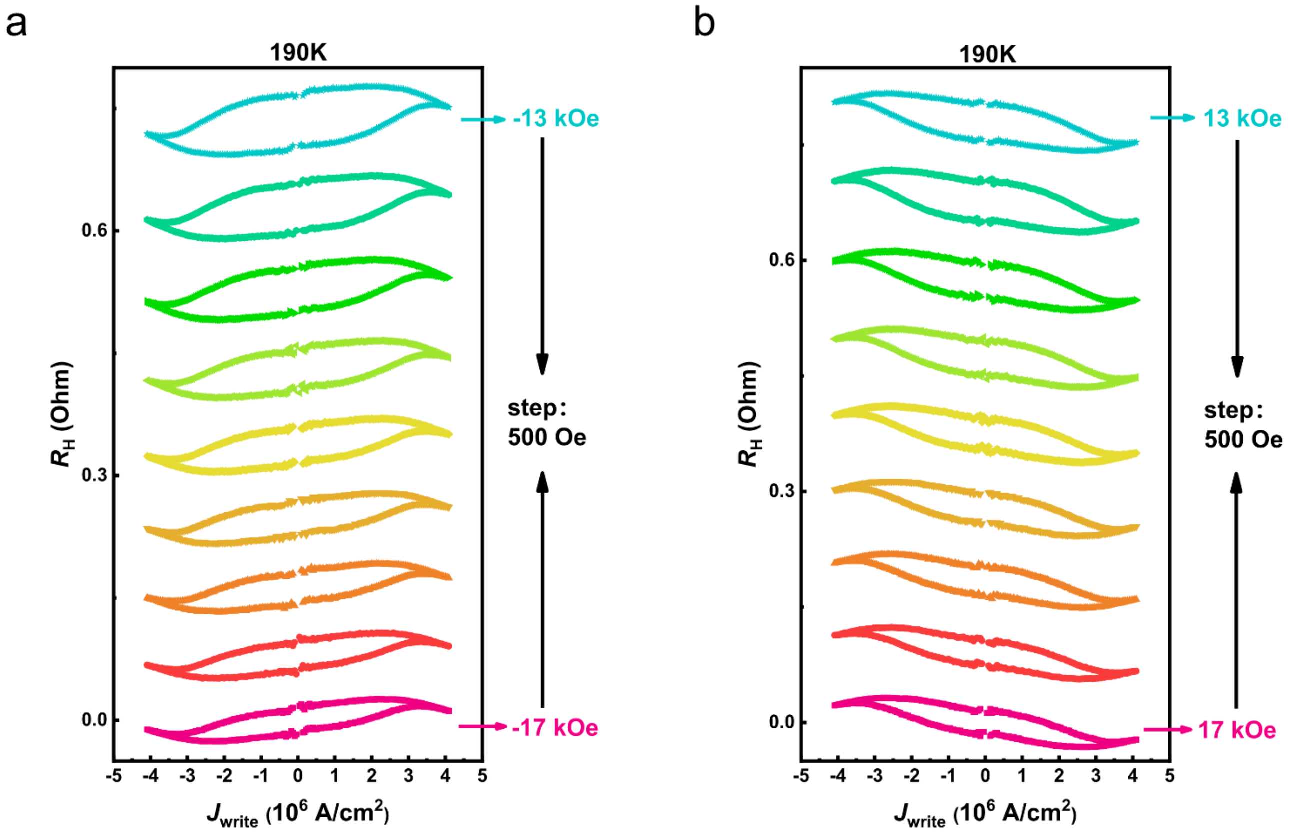

Figure S3 | Spin-orbit torque induced magnetization switching behaviors in Bi_2Te_3(8)/Fe_3GeTe_2(3) at 190 K. a, b SOT-induced magnetization switching behaviors at 190 K, which clearly show the relation between external in-plane field and switching current density for switching.

4. Temperature-dependent resistivity from Bi_2Te_3 and FGT thin films

To elucidate the resistivity properties of Bi_2Te_3 and FGT, we performed transport measurements by the physical property measurement system. The temperature-dependent resistivity from Bi_2Te_3 and FGT is shown in Fig. S4. It shows that the resistivity value of the 5nm-FGT increases with the decrease of temperature, which is due to the surface disorder caused by the thin film sample exposed to the air[3,4]. The R-T curve shows more insulating behaviors, which is in sharp contrast with the metal characteristic of thick FGT in Fig. S4b. It further proves that the thickness dependence will affect the interface of the heterostructure. Fig. S4c displays the typical metallic behavior of 8nm-Bi_2Te_3 from 100 K to 300 K.

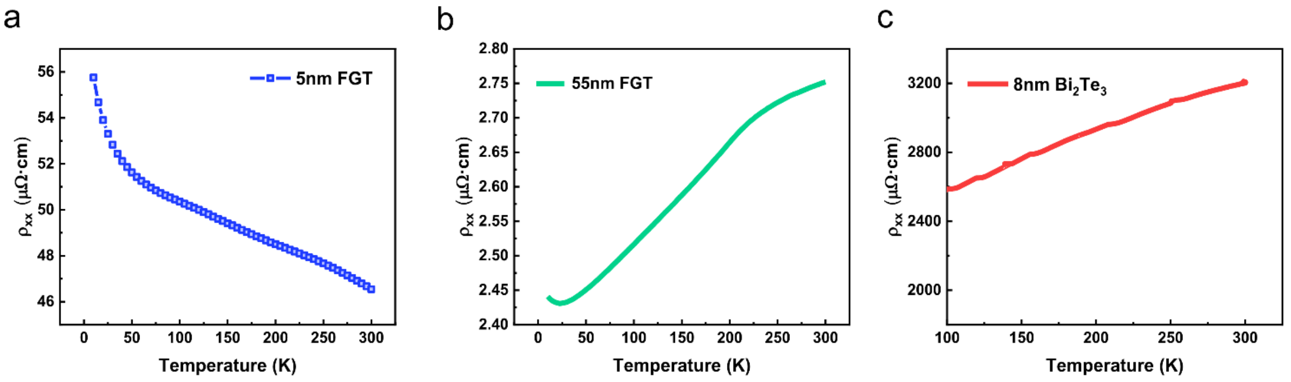

Figure S4 | Resistivity-temperature curves of Bi_2Te_3 and FGT thin films. a, b Temperature-dependent resistivity of the Bi_2Te_3 and FGT device, respectively.

5. Magnetotransport properties of Bi_2Te_3/FGT heterostructure

To prove the high interfacial thermal conductance in the Bi_2Te_3(8)/FGT(4) structure, the sweeping in a large range of in-plane magnetic field was carried out for observing the R_{xy} changing behavior under different currents, and typical results were shown in Fig. S5. It is found that the loops are almost the same even under large current of 1.5 mA, which prove the high thermal conductance of the Bi_2Te_3/FGT interface.

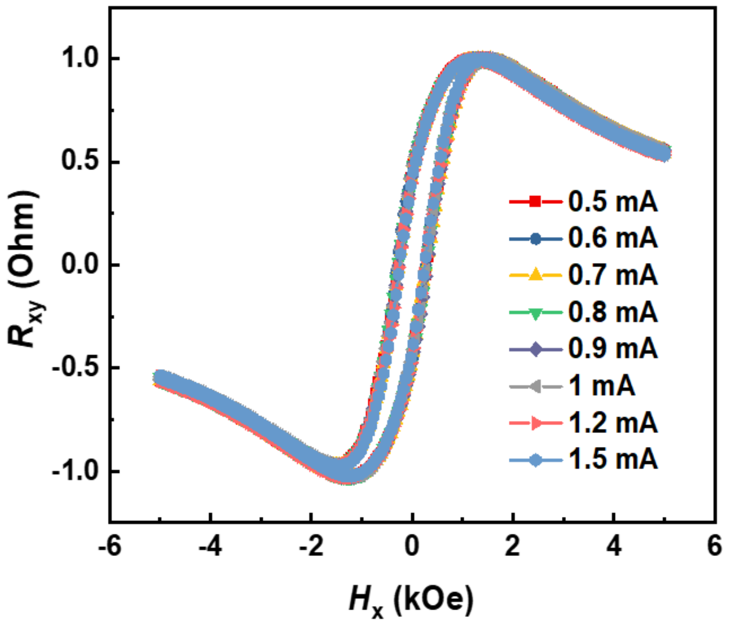

Figure S5 | Transport properties of the Bi_2Te_3/FGT heterostructures. In-plane field-dependent R_{xy}

of the Bi_2Te_3/FGT device under different currents.

6. Harmonic Hall voltage measurement and SOT efficiency calculation in Bi_2Te_3(8)/FGT(4)

To accurately describe the damping-like SOT efficiency through a harmonic Hall measurement, two methods are usually employed for fitting the data, including large-field power-law fitting and small field derivation fitting[5,6]. Formula (1) is the extended Landau-Lifshitz-Gilbert (LLG) equation that includes two spin torque terms. Here, the $\hat{m}$ is a unit vector of the magnetization direction, $\hat{p}$ is the average spin direction of the electrons diffusing into the magnetic layer, α is the Gilbert damping constant, γ is the gyromagnetic ration, $-\frac{\partial E}{\partial \vec{M}}$ is the current independent effective magnetic fields, a_J is the damping-like (Slonczweski-Berger) term and b_J is the field-like term[5]. To avoid the thermal-related phenomena from anomalous Nernst effect[7,8], we perform the fitting on the Bi_2Te_3(8)/FGT(3) sample using the former case following formula (2), where the equilibrium direction of the magnetization vector is pointing almost into the in-plane direction. Further, the low field values of $V_{2\omega}$ provide information of b_J, whereas the higher-field values give a_J for in-plane magnetic anisotropy (IMA). Therefore, for samples with less strong perpendicular magnetic anisotropy (PMA), a large field fitted damping-like term is more accurate.

$$\frac{\partial \hat{m}}{\partial t} = -\gamma \hat{m} \times \left(-\frac{\partial E}{\partial \vec{M}} + a_J(\hat{m} \times \hat{p}) + b_J \hat{p}\right) + \alpha \hat{m} \times \frac{\partial \hat{m}}{\partial t} \quad (1)$$

$$R_{2\omega} = \frac{R_A}{2}\frac{H_{DL}}{|H_x| - H_k} + R_p \frac{H_{FL}}{|H_x|} + R_{SSE+ANE}\frac{H_x}{|H_x|} + R_{offset} \quad (2)$$

$$H_{DL(FL)} = -2\frac{\mathrm{d}V_{2\omega}/\mathrm{d}H_{\mathrm{x(y)}}}{\mathrm{d}^2 V_{1\omega}/dH_{\mathrm{x(y)}}^2} \quad (3)$$

However, in absence of the thermal-related effect, the latter case that followed formula (3) should be more accurate for estimating SOT efficiency, since the former case still included field-like effective term. The formula (3) can make it clear that H_x and H_y represent a_J and b_J, respectively. The prerequisite condition required by this equation is that the direction of equilibrium magnetization

doesn't deviate significantly from the z-axis[5]. Therefore, we employed the small field derivation fitting method for the Bi_2Te_3(8)/FGT(4) heterostructure to evaluate the damping-like SOT efficiency, since this sample shows negligible thermal-related effect and strong PMA feature at low temperature. Compared to formula (3), the presence of field-like term in formula (2) potentially promotes the magnetization switching and affects the damping-like component. Meanwhile, the domain wall reflection at sample edges produces the possible phenomenon of anomalous SOT[9,10]. Following formula (3), we performed the in-phase first and out-of-phase second harmonic Hall measurements on the Bi_2Te_3(8)/FGT(4) heterostructure under different alternating currents and temperatures, as shown in Fig. S6. It clearly demonstrates the first and second harmonic signals from 100 K to 250 K, and the fitting results for damping-like SOT efficiency are shown in the main text.

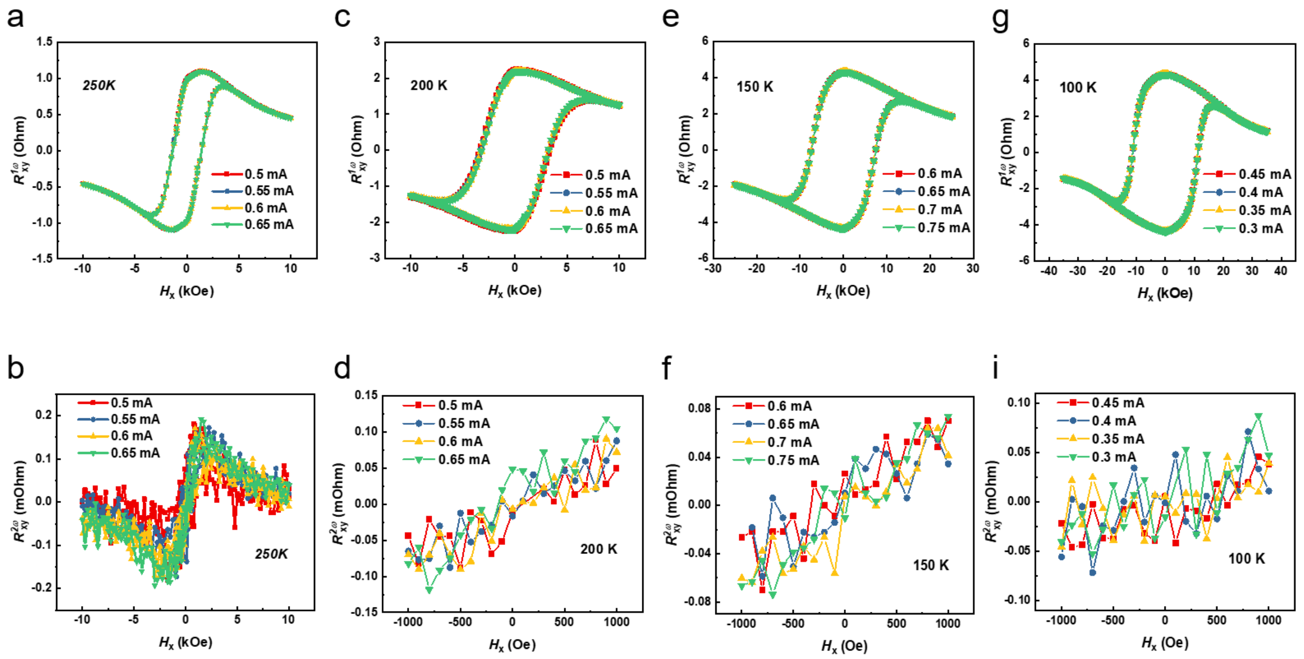

Figure S6 | Harmonic Hall voltage measurement and SOT efficiency calculation of the Bi_2Te_3(8)/FGT(4) heterostructure. The first harmonic Hall resistance signals (a), (c), (e), (g), and the second harmonic Hall resistance signals (b), (d), (f), (i) along the x-direction for the spin-up (down) states.

7. Estimation of Fermi level in Bi_2Te_3(8) structure at different temperature

To quantitatively analyze the position of the E_F level and its influence on the SOT switching, the following equation was employed[11]:

$$E_F = \hbar^2/(2m^*)\,(3\pi^2 n)^{2/3} \quad (3)$$

where $\hbar$ is the reduced Plank constant, $m^* \approx 0.15m_0$ is the effective mass and n is the bulk carrier density. Hence, we further conduct the Hall measurements to estimate the bulk carrier density n at different temperatures as shown in Fig. S7a. The illustration of the inset elucidates the band structure of Bi_2Te_3 and the location of the E_F, which more explicitly reveals the source of Bi_2Te_3 carriers[12]. The E_F is located in the bulk conduction band, above the Dirac-cone of surface state[13]. The fact that TI with reduced bulk conductance leads to a higher SOT efficiency suggests that the TSS renders significant contributions to the efficient SOT. Figure. S7b displays the temperature-dependent E_F values for estimating its position related to the bulk conduction band and the inset presents the simplified diagram.

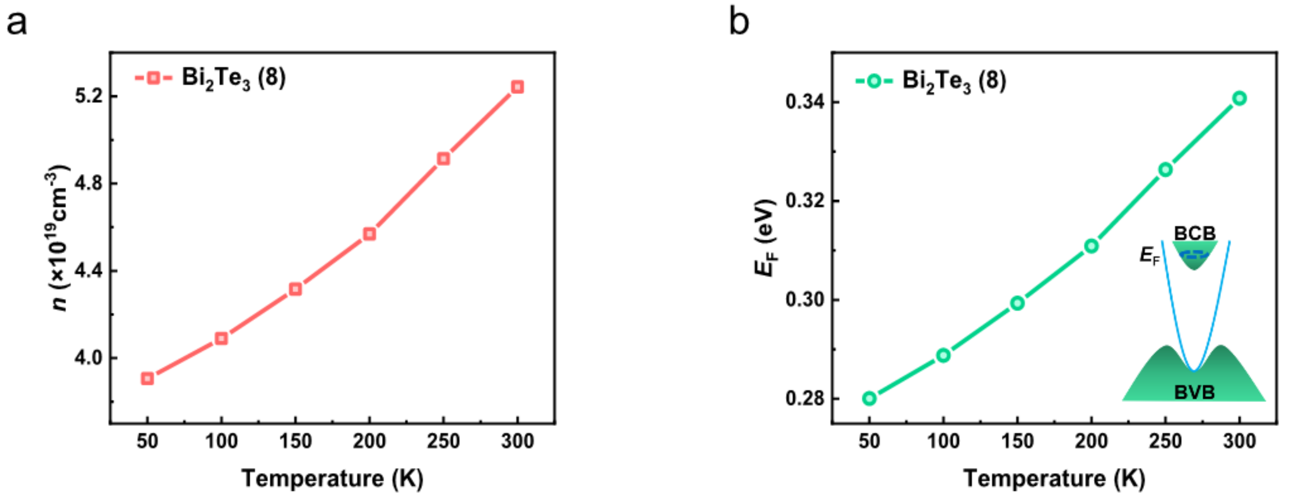

Figure S7 | Estimation of Fermi level in Bi_2Te_3(8) structure at different temperatures. The bulk carrier density as a function of temperature in Bi_2Te_3 structure. The inset shows the schematic of the Fermi level position.

8. Harmonic Hall voltage measurements in Bi_2Te_3(6)/FGT(4) and Bi_2Te_3(10)/FGT(4)

heterostructures at 300K

To comprehensively analyze the physical mechanism of realizing SOT switching, we further prepared the Bi_2Te_3(6)/FGT(4) and Bi_2Te_3(10)/FGT(4) heterostructures and carried out the harmonic measurements. Figure S8 displays the in-plane magnetic field-dependent first and second harmonic Hall resistance signals from these two samples, meanwhile the signal from Bi_2Te_3(8)/FGT(4) was also demonstrated as a reference. The value of the H_{DL}/J_{write} for Bi_2Te_3(6)/FGT(4) and Bi_2Te_3(10)/FGT(4) are ~69.32 Oe per MA/cm^2 and ~2.04 Oe per MA/cm^2, respectively, and the calculated SOT efficiency are correspondingly determined to be ~2.69 and ~0.08 at 300 K. Hence, the significantly enhanced SOT efficiency through reducing conductive bulk state proves the significant role of surface state. The above results provide insights for distinguishing the surface and bulk contribution that realizes the SOT switching.

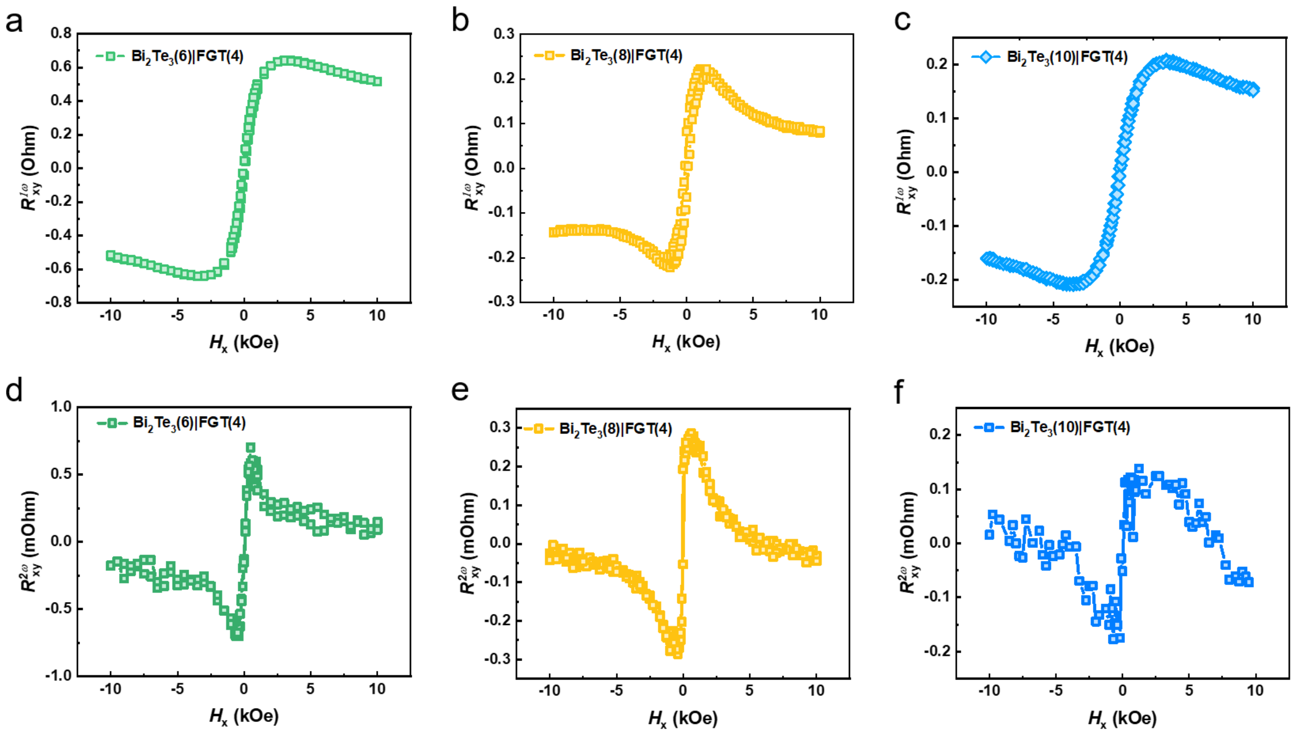

Figure S8 | Harmonic Hall voltage measurements in Bi_2Te_3(6)/FGT(4) heterostructure at 300K. The first harmonic Hall resistance signals (a), (b), (c) and the second harmonic Hall resistance signals

(d), (e), (f) along the x-direction of the external magnetic field with different Bi_2Te_3 thickness.

Supplementary References:

1. Kou, X. *et al.* Interplay between Different Magnetisms in Cr-Doped Topological Insulators. *ACS Nano* **7**, 9205–9212 (2013).
2. Wang, H. *et al.* Above Room-Temperature Ferromagnetism in Wafer-Scale Two-Dimensional van der Waals Fe_3GeTe_2 Tailored by a Topological Insulator. *ACS Nano* **14**, 10045–10053 (2020).
3. Tan, C. *et al.* Hard magnetic properties in nanoflake van der Waals Fe_3GeTe_2. *Nat. Commun.* **9**, 1–7 (2018).
4. Deng, Y. *et al.* Gate-tunable room-temperature ferromagnetism in two-dimensional Fe_3GeTe_2. *Nature* **563**, 94–99 (2018).
5. Hayashi, M., Kim, J., Yamanouchi, M. & Ohno, H. Quantitative characterization of the spin-orbit torque using harmonic Hall voltage measurements. *Phys. Rev. B* **89**, 144425 (2014).
6. Schulz, T. *et al.* Effective field analysis using the full angular spin-orbit torque magnetometry dependence. *Phys. Rev. B* **95**, 224409 (2017).
7. Shao, Q. *et al.* Strong Rashba-Edelstein Effect-Induced Spin–Orbit Torques in Monolayer Transition Metal Dichalcogenide/Ferromagnet Bilayers. *Nano Lett.* **16**, 7514–7520 (2016).
8. Avci, C. O. *et al.* Interplay of spin-orbit torque and thermoelectric effects in ferromagnet/normal-metal bilayers. *Phys. Rev. B* **90**, 224427 (2014).
9. Legrand, W., Ramaswamy, R., Mishra, R. & Yang, H. Coherent Subnanosecond Switching of Perpendicular Magnetization by the Fieldlike Spin-Orbit Torque without an External Magnetic Field. *Phys. Rev. Appl.* **3**, 64012 (2015).
10. Yoon, J. *et al.* Anomalous spin-orbit torque switching due to field-like torque-assisted domain wall reflection. *Sci. Adv.* **3**, 4 (2017).
11. Brahlek, M., Koirala, N., Bansal, N. & Oh, S. Transport properties of topological insulators: Band bending, bulk metal-to-insulator transition, and weak anti-localization. *Solid State Commun.* **215–216**, 54–62 (2015).
12. Suh, J. *et al.* Fermi-level stabilization in the topological insulators Bi_2Se_3 and Bi_2Te_3: Origin of the surface electron gas. *Phys. Rev. B* **89**, 115307 (2014).
13. Chen, Y. L. *et al.* Experimental Realization of a Three-Dimensional Topological Insulator, Bi_2Te_3. *Science* **325**, 178–181 (2009).
